# Key epidemiological indicators and spatial autocorrelation patterns across five waves of COVID-19 in Catalonia

**DOI:** 10.1038/s41598-023-36169-2

**Published:** 2023-06-15

**Authors:** Francesc Belvis, Alberto Aleta, Álvaro Padilla-Pozo, Juan-M. Pericàs, Juan Fernández-Gracia, Jorge P. Rodríguez, Víctor M. Eguíluz, Charles Novaes De Santana, Mireia Julià, Joan Benach, the COVID-SHINE group, Núria Benach, Lucinda Cash-Gibson, Carles Delclós, Mariana Gutiérrez-Zamora, Eliana Martínez-Herrera, John Palmer, Diego-F. Rojas-Gualdrón

**Affiliations:** 1 Research Group on Health Inequalities, Environment, and Employment Conditions (GREDS-EMCONET), Department of Political and Social Sciences, Universitat Pompeu Fabra, 08005 Barcelona, Spain; 2 Johns Hopkins University-Universitat Pompeu Fabra Public Policy Center (JHU-UPF PPC), 08005 Barcelona, Spain; 3 Institute for Biocomputation and Physics of Complex Systems (BIFI), University of Zaragoza, 50018 Zaragoza, Spain; 4 Department of Sociology, Cornell University, Ithaca, New York, USA; 5 Liver Unit, Internal Medicine Department, Vall d’Hebron University Hospital, Vall d’Hebron Institute for Research, CIBERehd, 08035 Barcelona, Spain; 6 Infectious Disease Department, Hospital Clínic, 08036 Barcelona, Spain; 7 Instituto de Física Interdisciplinar Y Sistemas Complejos IFISC (CSIC-UIB), 07122 Palma de Mallorca, Spain; 8 Instituto Mediterráneo de Estudios Avanzados IMEDEA (CSIC-UIB), 07190 Esporles, Spain; 9 ESIMar (Mar Nursing School), Parc de Salut Mar, Universitat Pompeu Fabra-Affiliated, 08003 Barcelona, Spain; 10 SDHEd (Social Determinants and Health Education Research Group), IMIM (Hospital del Mar Medical Research Institute), 08005 Barcelona, Spain; 11 Ecological Humanities Research Group (GHECO), Universidad Autónoma de Madrid, 28049 Madrid, Spain; 12 Departament de Geografia, Universitat de Barcelona, 08001 Barcelona, Spain; 13 Pompeu Fabra University–UPF Barcelona School of Management (UPF-BSM), 08005 Barcelona, Spain; 14 Institut de Govern i Polítiques Públiques, Universitat Autònoma de Barcelona, 08193 Bellaterra, Spain; 15 Research Group of Epidemiology, National School of Public Health “Héctor Abad Gómez”, University of Antioquía, Medellín, Colombia; 16 Departament de Ciències Polítiques i Socials, Pompeu Fabra University, 08005 Barcelona, Spain; 17 Faculty of Medicine, CES University, Medellín, Colombia

**Keywords:** Epidemiology, Statistics

## Abstract

This research studies the evolution of COVID-19 crude incident rates, effective reproduction number R(t) and their relationship with incidence spatial autocorrelation patterns in the 19 months following the disease outbreak in Catalonia (Spain). A cross-sectional ecological panel design based on n = 371 health-care geographical units is used. Five general outbreaks are described, systematically preceded by generalized values of R(t) > 1 in the two previous weeks. No clear regularities concerning possible initial focus appear when comparing waves. As for autocorrelation, we identify a wave’s baseline pattern in which global Moran’s I increases rapidly in the first weeks of the outbreak to descend later. However, some waves significantly depart from the baseline. In the simulations, both baseline pattern and departures can be reproduced when measures aimed at reducing mobility and virus transmissibility are introduced. Spatial autocorrelation is inherently contingent on the outbreak phase and is also substantially modified by external interventions affecting human behavior.

## Introduction

The study of spatial autocorrelation phenomena has proved useful in epidemiological decision-making and planning concerning COVID-19^1,2^. As in other transmissible diseases, spatial clustering of COVID-19 cases arises from a combination of first-order processes (similarity of neighboring areas concerning the distribution of risk factors for the disease) and second-order processes of contagion and diffusion^3^, although depending on the epidemiological parameters such as the incubation period, the spatial patterns might be less predictable^4^. In both cases, it is reasonable to expect that spatial adjacency phenomena between areas will appear, whether defined as geographical contiguity, distance or similar criteria. It can be argued that spatial proximity is not an optimal criterion in the case of humans, given that modern transportation facilities and socioeconomic dependencies between areas are often more determinant for human interactions than pure geographical distance. Indeed, hierarchical diffusion from one location, usually more central, to other locations not necessarily close in space has been described in relation to COVID-19^5^. These hindrances can be potentially overcome by incorporating adjacency based on mobility into modeling.

On the other hand, dynamics, i.e., evolution over time, is a critical dimension in the COVID-19 research field. Two major cyclical aspects of coronavirus cases have been identified: the “weekend effect” and “waves”^6^, the latter being of most interest for epidemic control. Already the early Imperial College report of March 16, 2020^7^ foresaw a scenario of successive epidemic cycles in a country, driven by strengthening and relaxation of Non-Pharmaceutical Interventions (NPIs) that could not be permanently maintained because of their damaging consequences on social and economic life. The prediction of successive waves has been fulfilled, although we know now that not only the relaxation of government policies is important but also changes in social behavior, the importation of infected cases^8^, and temperature^9^. Furthermore, the emergence of virus mutations^10^ and the effects of vaccination among others have become crucial in a further stage of the pandemic. Despite the ubiquity of the term, a standard operational definition of a “wave” does not exist^11,12^. However, this does not preclude the usefulness of the concept, nor the identification of waves in practice, mainly through the peaks and valleys in the epidemics indicators such as the incidence crude rates or the effective reproduction number R(t).

Spatial autocorrelation phenomena have hardly been considered in relation to the epidemic cycle looking specifically for a relationship between them. There is empirical evidence that the autocorrelation significantly varies within and across waves in the short^13,14^ and long term^15–17^. However, most of these analyses focus on looking for substantial information concerning the clustering of incident cases. Temporally high autocorrelation values were observed in Malaysia and this was interpreted by the authors as informing “the spatial dynamics of the initial outbreak, in addition to the incidence rate and time-varying reproduction number”^15^. Nevertheless, so far there is scarce evidence guiding how spatial autocorrelation should be interpreted as the epidemic cycle evolves.

This study aims to fill this gap by analyzing global autocorrelation evolution in a long COVID-19 pandemic period in the region of Catalonia (Spain). Our objectives are: (1) to describe the five COVID-19 epidemic waves in terms of incidence and effective reproduction number R(t); (2) to analyze and compare the spatial autocorrelation patterns produced in the successive waves according to several proximity and mobility criteria; and (3) to produce a simulation model aimed to unravel the eventual mechanisms generating the observed autocorrelation patterns.

## Methods

### Study design

This is a cross-sectional panel ecological design studying the autonomous region of Catalonia, Spain. At the beginning of 2020, the population was n=7,653,845 inhabitants, which was assumed to be the population at risk throughout the study period. Geographically, Catalonia has a triangular-shaped surface of 32,000 km^2^ and the maximum road distance between its furthermost points is about 360 km. The spatial study units are the Basic Health Areas (BHA) used by the Catalan Health Department to organize primary healthcare services. A BHA encompasses a reference territory of a primary health care team and its population. This administrative division tends to make population volume roughly equal, which leads to wide differences in geographical extension between rural and urban areas, while protecting against extremely high or low crude rates^18^. The number of BHA in this study was n=371 according to the 2018 official geographical definition. BHAs are nested within nine broader Health Regions. Population distribution by BHA was also obtained from the Catalan Health System.

### Crude incidence rate and R(t)

According to Catalan Health Department Guidelines, incident counts are defined as “accumulated confirmed cases” of SARS-CoV-2 infection by means of: (a) positive polymerase chain reaction tests (PCR) and/or rapid antigen tests (RAT) performed according to varying screening strategies; (b) cases confirmed by epidemiological staff; (c) probable cases, where tests have not been conclusive but are given a high probability of being positive; and (d) serologically confirmed cases by means of ELISA and rapid tests which indicate the patient’s immune status^19^.

The daily incident counts per BHA in the region were retrieved from open surveillance data maintained by the Catalan autonomous government. Epidemics was assumed to start on day March 1st 2020 and residual cases previous to that date were given this value. Daily cases were aggregated later by groups of 7 days and the result was divided by the BHA population, thus yielding comparable brute incidence rates per 100,000 inhabitants across 85 weeks from the beginning of the pandemic. The aggregation by less than seven days was discarded as it resulted in very irregular patterns of incident cases at the BHA level, due to the weekend effect and probably linked to social conditioning in data reporting^6^. Please note that weekly aggregation implies that the limits of the waves in our study may not coincide exactly with the official daily limit of the Ministry of Health. On the other hand, the number of cases in the first weeks of the epidemic was largely underreported: a country-wide seroprevalence study carried out in Spain between April 27 and May 11 2020 showed that the overall prevalence was 10 times larger than the number of detected cases^20^.

A second indicator of the pandemic, the effective reproduction number R(t), was also considered in this research. R(t) represents the average number of new infections caused by each new infectious case. If its value is larger than 1, the outbreak will be growing. In contrast, a value smaller than 1 means that the incidence is decreasing. As such, it provides a very simple way of determining when an intervention is enough to contain an outbreak—if R(t) is smaller than 1, the outbreak will be smaller every day. For this reason, it is usually used to determine the effectiveness of government interventions, although in retrospective rather than for real-time monitoring^21–23^ because of real-time data quality issues^24,25^.

To estimate R(t) value we used a methodology that relies on non-stationary Gaussian processes^26^. We set the generation time to the one estimated in Ganyani et al.^27^ and the incubation period presented in Lauer et al.^28^. Besides, we assumed a three-day notification delay following the recommendation of the Spanish Ministry of Health^29^. Weekly estimates of the time-varying R(t) were then estimated by taking the mean of each daily expected R(t) in each BHA for each week.

### NPIs, mobility between BHAs and vaccination

In addition to qualitative information concerning the main NPIs affecting Catalonia, we have also employed the Stringency Index, a composite measure that records the strictness of government antiCOVID-19 NPIs around the world^30^, and specifically for Spain. Mobile phone data have been used worldwide as a proxy for the general movement of the population and to understand the impact and compliance of the population to NPIs^31^. In this research, an estimation of mobility between BHA during the period was built from publicly available mobile phone data from February 22, 2020 to May 9, 2021 made public by the Spanish Ministry of Transports, Mobility and Urban Agenda. The n = 881 mobility areas in Catalonia of the original study were superposed to the BHA polygons thread, and the weekly number of travels from an origin BHA ‘i’ to a destination BHA ‘j’ was approximated by means of assumptions of area (km^2^) proportionality. Finally, weekly aggregated levels of vaccination data for Catalonia and the study period have been also obtained for control purposes.

### Statistical analysis

Heatmaps of incidence rates and R(t) organized by Health Region and BHAs across the period were constructed to show the quantitative course of the pandemic. Basic contextual data such as main health-policy measures enacted by the Spanish and Catalan governments (provided by their respective COVID-19 online information services) are also reported. General global Moran’s I was used to examine if COVID-19 weekly incident rates were spatially autocorrelated. Moran’s I values range from − 1 to + 1. If the Moran’s I value is positive and significant, there is a clustering of COVID-19 rates within the “proximal” geographic areas (BHA in this case). The significance level of Moran’s I test was established at p=0.05.

Three main criteria of adjacency between BHAs were considered: (1) shared border according to a “queen” criteria; (2) geographical distance between BHAs centroids; and (3) travels between BHAs estimated from mobile phone data, as explained above. In this case, external movements to and from Catalonia were excluded. Weights matrices were constructed accordingly and the Moran’s I values obtained according to the three criteria were analyzed and compared. Exploratory linear regression models predicting crude incidence rates from their temporal and spatiotemporal lags were adjusted to check the impact of the epidemic trend on the spatial dependence (Moran’s I) of the residuals. Finally, additional linear regression models predicting local R(t) from lagged Stringency Index and population vaccination levels were fitted to test the possible influence of these factors.

### Autocorrelation simulation

Given the multiple NPIs imposed by governments throughout the pandemic, particularly in terms of limiting mobility during 2020 waves, the spatial patterns may deviate from the expected ones in an unmanaged outbreak. We therefore performed stochastic simulations of an epidemic spreading on a synthetic population of Catalonia to assess potential typical patterns during a “non-intervened wave”. In particular, we build a metapopulation system composed of multiple subpopulations, each of them representing one BHA^32,33^. Each day, a certain fraction of the population in each BHA would move to other subpopulations. The fraction of travellers and the destination was extracted from the data provided by the Spanish Ministry of Transports, Mobility and Urban Agenda, so that it resembles the real mobility of the population before the pandemic started. Within each subpopulation, we implemented a homogeneous mixing SEIR model^34–37^ characterized using the natural history of the first variant of COVID-19 that spread throughout the world in early 2020^38^.

The purpose of the model was to understand what is the evolution of the spatial autocorrelation that one would expect under different circumstances. As such, we first simulated the extreme situation in which no interventions are set in place, nor the behavior of people is modified (henceforth, baseline scenario). Then, we also simulated three different scenarios: one with 100 times less mobility (*low mobility*); one with 60% less transmissibility, representing the multiple measures aimed at reducing its value such as social distancing, masking, closure of certain venues… (*reduced transmissibility*); and one in which the transmissibility is reduced and in the third week of the outbreak mobility is completely closed (*reduced transmissibility* + *closed mobility*). Finally, we measured the evolution of crude incidence rates and Moran’s I as a function of time in all these scenarios.

### Software

Data retrieving, manipulation and statistical analysis were performed within the R environment v. ‘4.2.1’. R(t) general and local estimations were obtained using the package EpiNow2, while autocorrelations were calculated using the spdep package. The stochastic simulations were implemented in C and analyzed using R, as with the real data. All Figures in both the paper and Supplementary information were created by the authors using the ggplot2 package v. ‘3.4.0’, https://ggplot2.tidyverse.org.

## Results

### Description of the waves

**Figure 1:**
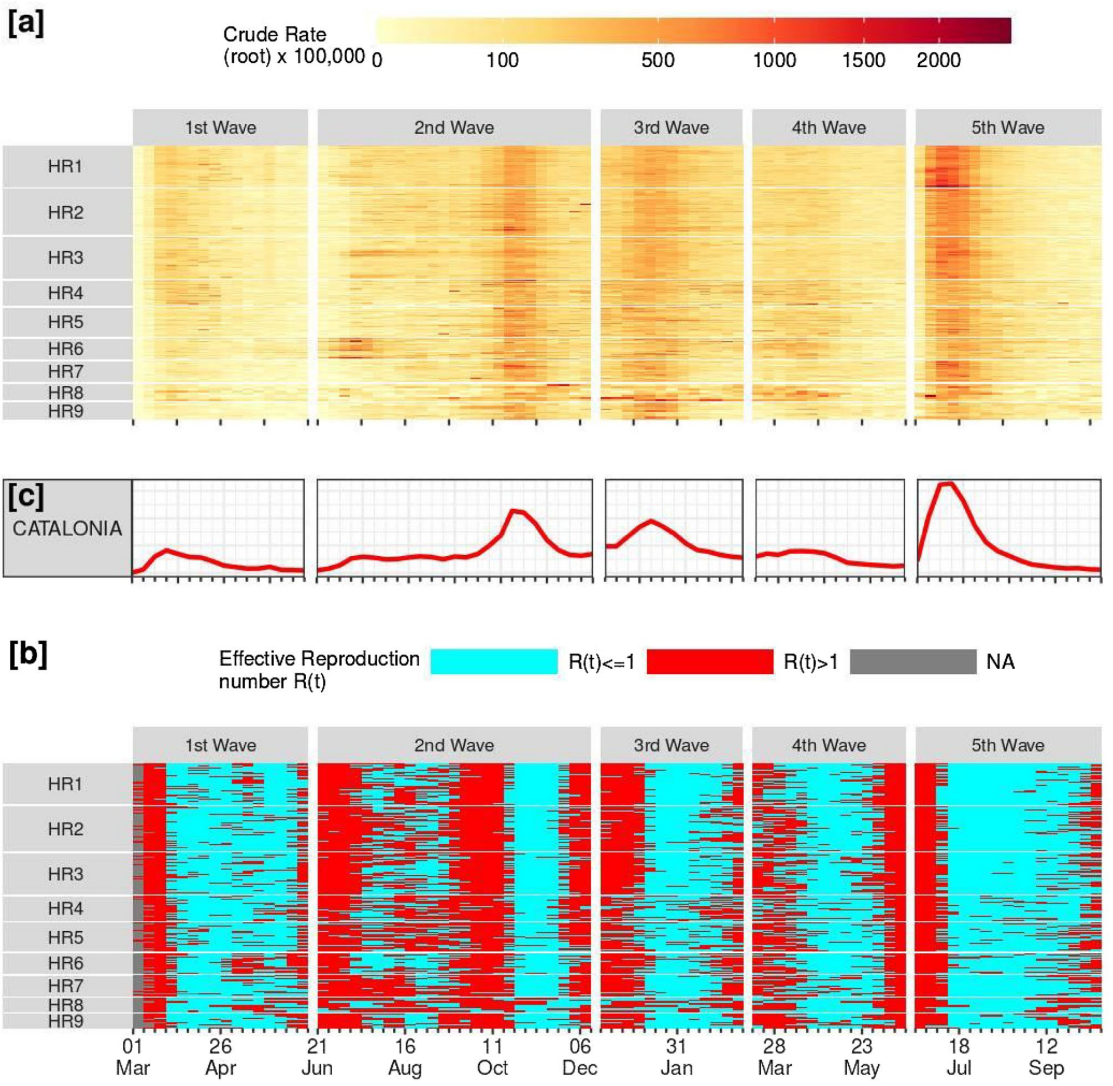
Catalonia. Evolution of crude (root) incidence rates (**a**) and effective reproduction number R(t) (**b**) by BHAs and Health Region, plus marginal incidence crude rates (**c**). HR1: Barcelona, HR2: Metropolitan North, HR3: Metropolitan South, HR4: Central Catalonia, HR5: Province Capital, North (Girona hinterland), HR6: Province Capital, East (Lleida hinterland), HR7: Province Capital, South (Tarragona hinterland), HR8: Rural Northeast, HR9: Rural South. Note: this figure was created by the authors within the R environment using the ggplot2 package v. ‘3.4.0’, https://ggplot2.tidyverse.org.

During the timespan of this study the COVID-19 epidemic in Spain was officially divided into five waves of contagion according to inflection points in the evolution of the cumulative incidence of cases per 100.000 inhabitants in the prior 14 days. These approximately correspond with Catalan evolution and are assumed as waves in this research. Heatmap in Fig. 1a shows the weekly evolution of incidence rates by Health Region, BHA and the waves.

The *first wave* was considered to start on March 1st 2020 and was characterized by the beginning of community transmission, infra detection of cases, pressure on the health system, high mortality rates, and the general lockdown as a response, which was enforced in Spain on 15th March (3rd week in Fig. 1a). As a result, incidence fairly decreased almost everywhere and on June 21st the general lockdown was finished^39^ (112 days in total). A seroprevalence study carried out by the Spanish Ministry of Health^20^ estimated that 5.9% (5.0–7.1 95% CI) of the Catalan population had been infected during this first wave. Thus, most of the population was still susceptible. The *2nd wave*. In the new situation, residents could move around Spain with no restrictions, while COVID-19 health measures were decentralized to the regional autonomous governments that applied less intrusive NPIs. In Catalonia those included reduction/banning of outdoor social gatherings, compulsory use of masks and social distancing, closing of cafes and restaurants at midnight, as well as municipality-specific recommendations and enforcement of the tracing system. Two phases can be distinguished: in July, a significant rise in the general incidence rate began, which later stabilized until September 2020. However, the evolution of R(t), which was larger than one in several areas of the territory at this time, signaled that the epidemic was not completely under control (Fig. 1b). In late September, this unstable situation exploited and incidence rates fairly steeped to a maximum of 454.6 per 100,000 the week beginning October 18th. On October 25th the Spanish Government enforced a state of alarm (including a national curfew from 10 p.m. to 6 a.m. in the case of Catalonia)^40^ and the limitation of mobility in each autonomous region. These measures succeeded in inverting the curve and achieved a return to contagion rates similar to those of the summer *impasse* at the wave end on December 12th (168 days in total). The seroprevalence study estimated that by late November 2020, 11.6% (9.9–13.7 95% CI) of the Catalan population had been infected. Thus, the second wave was comparable with the first one in terms of infections but the global immunity of the population was still low. *3rd wave.* In terms of the incidence, a new outbreak was evident by the end of December, and in early December in terms of R(t). On January 4th, mobility was restricted within municipalities, while the state of alarm was in force during the entire wave. As incidence decreased, mobility restrictions were relieved from municipalities to districts (comarcas) on February 5th^36^ and the wave was considered finished on March 14^th^, 2021 (98 days in total). There were no further seroprevalence studies. Besides, vaccination started in early January for people in the most important risk groups. By the end of the wave, roughly 8% of the Catalan population had been inoculated with the first dose, and 3% were completely vaccinated (2 doses). *4th wave.* The overall incidence rate in this wave was low in comparison to the other waves (maximum of 158.6 per 100,000 the week beginning April 11th). Also, the epidemiological countermeasures adopted were much looser. Mobility across the Catalan territory became legal on April 23th^41^ and on May 9th the state of alarm was over, therefore mobility restrictions across Spain and the national curfew were lifted^42^. The incidence rate dropped until the end of this wave on June 19th, 2021 (98 days in total). From the beginning of May to the end of June 2021, an important effort was made by the government to increase vaccination coverage, and the percentage of the population vaccinated with a first dose increased from 25 to 50%. At this point, a large fraction of the population already had some kind of immunity, either natural after experiencing the infection or due to the vaccines. *5th wave.* This wave particularly affected younger age groups (who were mostly unvaccinated at the time). This outbreak reached the highest measured incidence rates of all the pandemic in Catalonia to that date (maximum of 654.3 per 100,000 the week beginning July 11th). After that, incidence fell steadily until 13th October 2021 (115 days at last). This wave also marks the arrival of the first variant of concern, the Alpha (B.1.1.7) variant, estimated to be between 40 to 80% more transmissible^43^. By the end of the wave, 75% of the Catalan population was completely vaccinated.

The “peaks” of the respective five waves can be appreciated as vertical reddish traces in Fig. 1a. Overall, vertical traces are much more evident than horizontal ones, which suggests that time is more relevant than space in explaining incidence evolution (at least at the weekly scale). However, some color clusters appear between Health Regions and BHAs within and between regions, which suggests there is some room for spatial clustering phenomena. For example, in 1st wave greater incidence BHAs seem located in HR1-HR2-HR3-HR4 (Barcelona and its metropolitan area together with Central Catalonia), while in 4th wave greater incidence BHAs are located in HR6 and HR8, both in the East of Catalonia. It can also be seen that in the fifth wave highest incidence BHAs belong to HR1 (Barcelona) (Fig. 1a). As for R(t) evolution (dichotomized at value >1, Fig. 1b), it is obvious that periods of generalized R(t) values over 1 (in red) precede in about two weeks a general incidence peak, exception made of 1st wave (probably because of data unavailability). The evolution of R(t) further indicates the importance of temporal correlations, given that most areas go over and under the threshold of R(t) = 1 around the same week.

**Figure 2:**
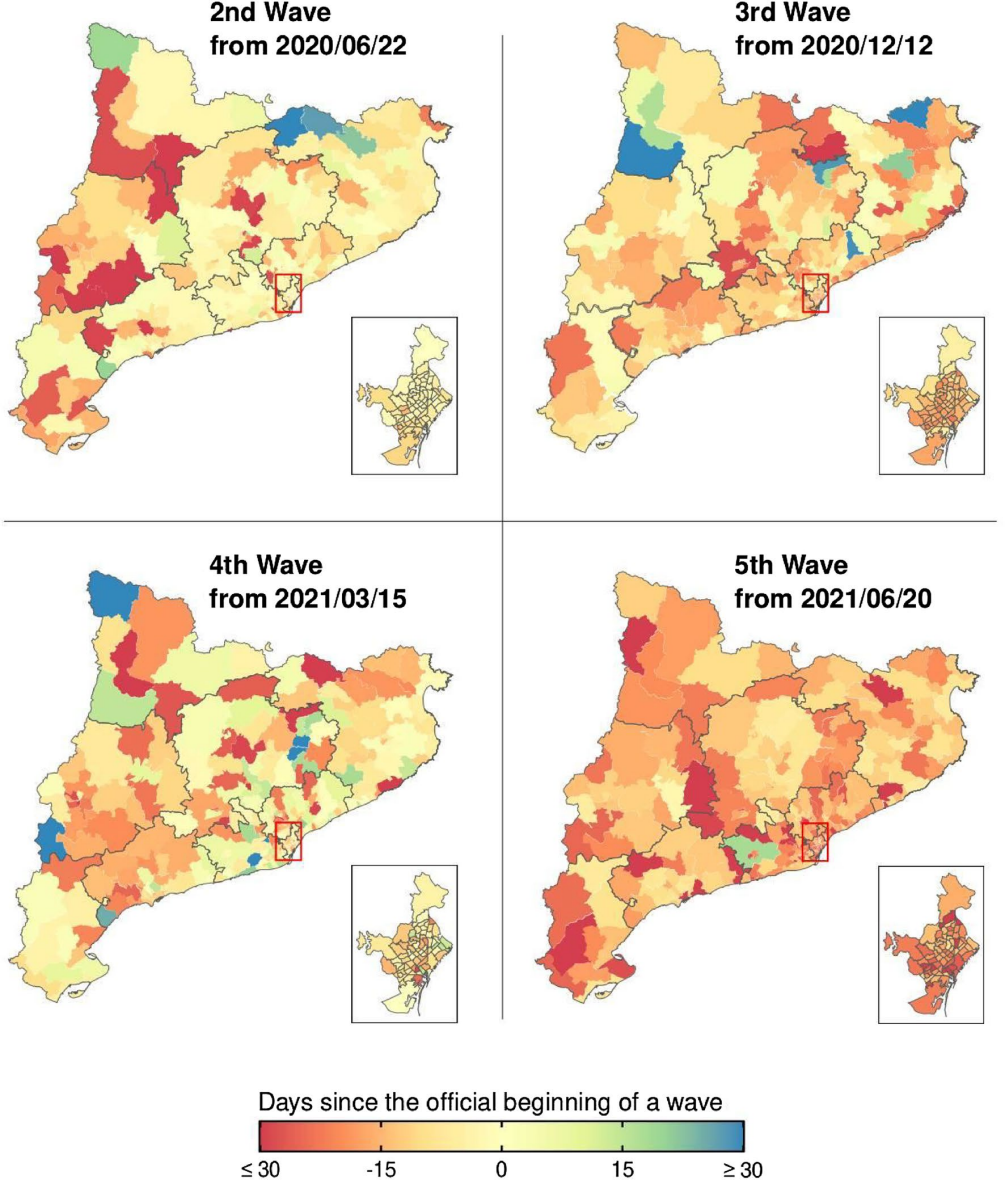
Catalonia. Number of days since the ‘official’ beginning of the wave until the value of R(t) increases above one. Negative values indicate that the incidence was already increasing in the region before the official declaration of a new wave, while positive values indicate that it started growing after it. The difference is measured as the number of days between the official date and the last time that R(t) was below one in case it was above one by the beginning of the wave, or between the official date and the first time that it increased above one if it was below one by the beginning of the wave. Note: this figure was created by the authors within the R environment using the ggplot2 package v. ‘3.4.0’, https://ggplot2.tidyverse.org.

To showcase the dissonance between outbreak growth and official wave declaration more clearly, the maps in Fig. 2 depict the number of days until R(t) becomes greater than 1 since the official beginning of the wave (i.e. number of days = 0), by BHA. In most parts of the territory, local outbreaks started several days or weeks before a new wave was officially established. Moreover, the BHAs where this occurred were specific to each wave and no clear pattern can be discerned by visual inspection.

### Spatial autocorrelation

**Figure 3:**
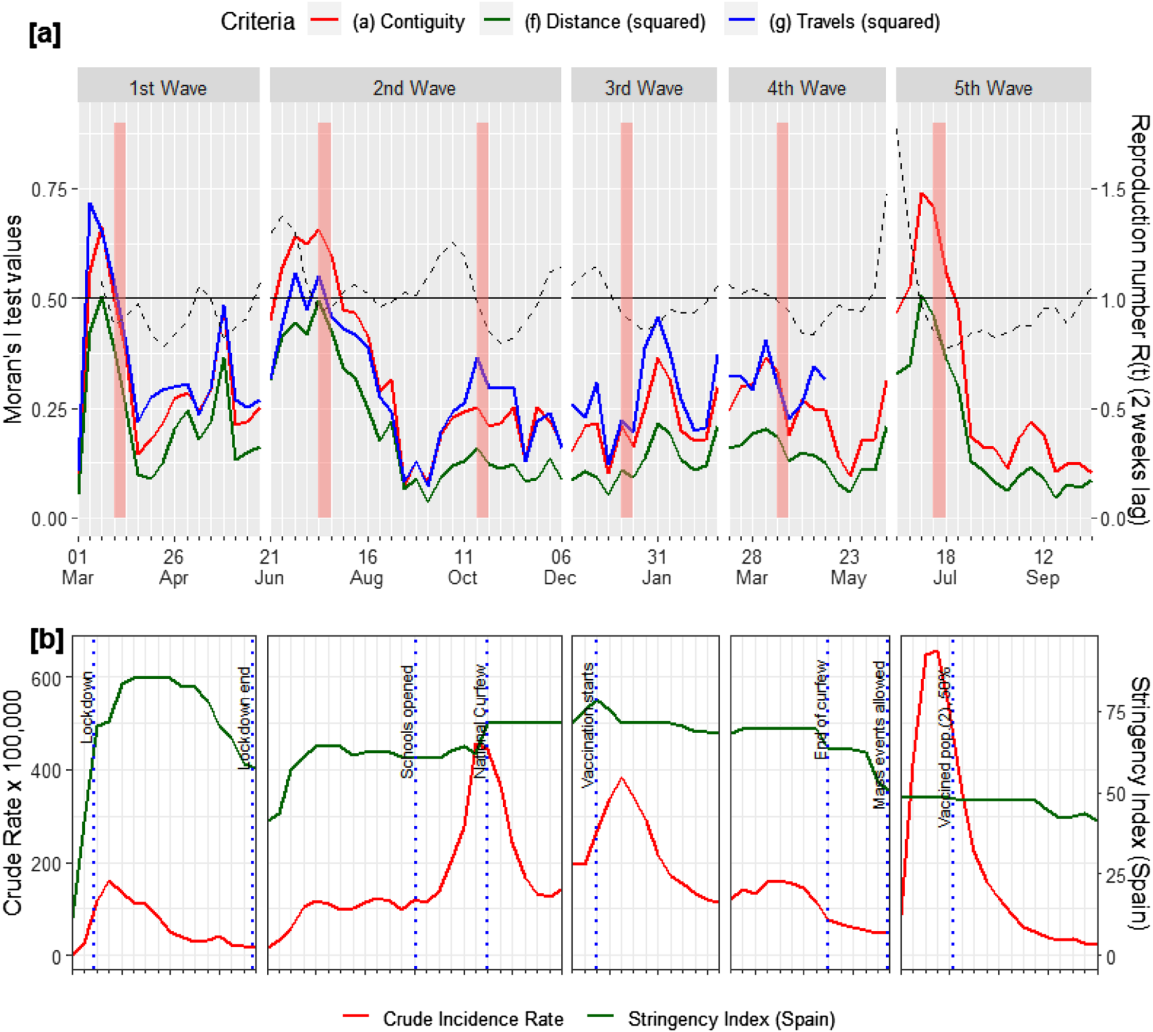
(**a**) Catalonia global spatial autocorrelation values (Moran’s I) of weekly crude incidence rates according to several criteria (left axis), and effective reproduction number R(t) (right axis); (**b**) Catalonia crude incidence rate evolution (left axis) and Stringency Index for Spain (right axis). Incidence peaks are marked as reddish vertical lines in (**a**). Note: This Figure was created by the authors within the R environment using the ggplot2 package v. ‘3.4.0’, https://ggplot2.tidyverse.org.

Figure 3 shows the global spatial autocorrelation (Moran’s I) values across the study period according to contiguity (shared border, “queen”), distance (inverse, squared) and mobility (inverse, squared) criteria. Centering on contiguity, we see that autocorrelation is always positive and significant, however, it varies wildly across and within waves from a minimum of M = 0.071 (p = 0.006) the week beginning 2020-09-06 (2nd wave), to a maximum of M = 0.738 (p < 0.001) the week beginning 2021-07-04 (5th wave). Note that, according to the previous results, we have considered the two phases of wave 2 independently. Comparing between waves, different autocorrelation patterns can be distinguished: for the 1st, 2nd (first phase) and 5th waves an initial large increase in Moran’s I is followed by a decay, then a second, smaller peak (this last feature is less clear depending on the wave) and a new decay.

The 2nd wave-2nd phase and the 4th wave appear to follow a similar pattern, although the Moran’s I values are quite lower (maximum M = 0.25, p < 0.001 and M = 0.36, p < 0.001 respectively). Finally, the 3rd wave clearly does not follow this pattern as Moran’s highest values do not occur during the first weeks, but during the second half of the wave.

It can also be seen that in each wave/phase, autocorrelation peaks coincide with no lag, or one-week lag, with incidence peaks. Autocorrelation seems also to be higher when lagged R(t) is > 1. In fact, the autocorrelation trend is moderately correlated with the global R(t) lagged by two weeks (r = 0.531). This is consistent with the former observation that values of R(t) > 1 precede the beginning of a new wave in about two weeks. The 3rd wave is, again, the clearest exception to these regularities.

Proximity criteria other than contiguity yield, generally speaking, lower global Moran’s I values (Figs. A1 and A2 in Appendices). This is for example the case of Health Sector membership criteria, a 29-categories administrative subdivision of Health Regions (Fig. A1 in Appendices). As for distances between centroids of BHAs, autocorrelation values increase when inverse squared distance weights are used. The trend series for inverse squared distances is shown in Fig. 3, although autocorrelation values improve if higher powers are used (data not shown). The same logic applies to mobility, although this criterion performs better than pure distance and slightly better than contiguity in the 3rd wave. In the case of mobility, matrices are different for each week and the inclusion of lagged matrices was a possibility, but without clear improvements in autocorrelation.

Overall, autocorrelation evolution over time is similar independently of the adjacency criteria used, as it is its relationship with R(t) that remains moderate around r = 0.5.

Lagged temporal and spatiotemporal crude incidence rates explain most of the variability of local incident rates (R^2^ = 0.601 and R^2^ = 0.564, respectively, see Table A1 in the Supplementary materials). Moreover, the observed global spatial dependency of crude incidence rates (contiguity criteria were used) is greatly reduced in the residuals of the former models (see Fig. A3 in the Supplementary materials). This indicates that the observed spatial dependency (Moran’s I) is largely influenced by the previous levels of the infection in the local areas. On the other hand, stringency measures together with vaccination levels have an important role in explaining the local R(t) variability (R^2^ = 0.323, see Table A2 in the Supplementary materials), which in turn determines the COVID-19 incidence trend. However, the difficulty of building a specific statistical model that relates the impact of NPIs on the spatial dependence of crude incidence rates should be noted.

### Simulation results

To understand the dynamics driving autocorrelation patterns and its variation, we perform numerical simulations of epidemic spreading in a synthetic version of Catalonia, while mimicking the initial number of focus of the disease (BHAs) and the implementation of several NPIs.

**Figure 4:**
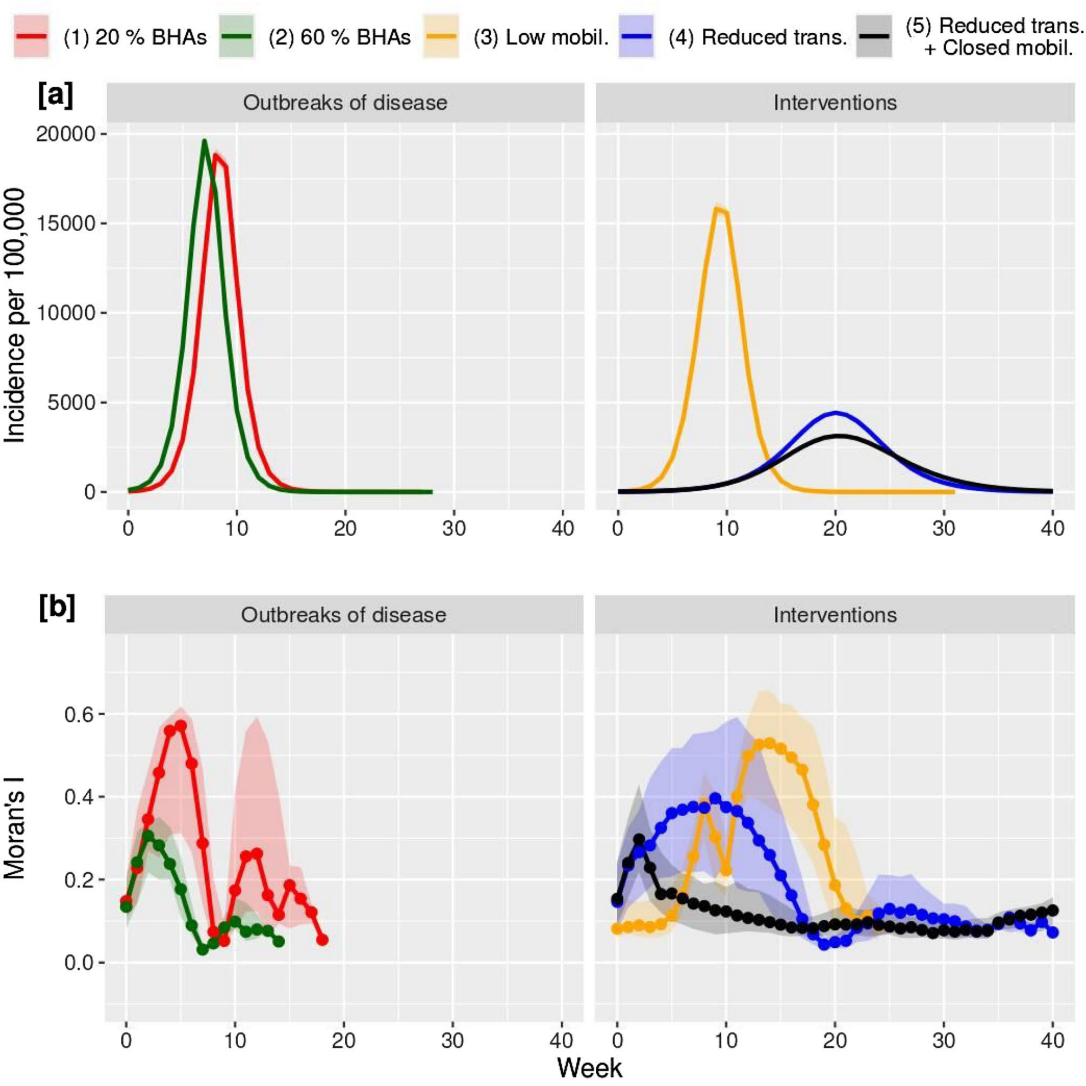
Incidence (**a**) and spatial autocorrelation (**b**) in numerical simulations of an epidemic cycle. Low (1) and high (2) number of outbreaks of disease (left) plus four different Non-Pharmaceutical Interventions scenarios (right) are considered: one with lower mobility across regions (3); one with lower transmissibility of the virus (4); and, lastly, one with lower transmissibility and a complete shutdown of mobility after the third week (5). Note: This Figure was created by the authors within the R environment using the ggplot2 package v. ‘3.4.0’, https://ggplot2.tidyverse.org.

In the baseline scenario, a localized start of the infection (the seed is to infect 0.1% of the population in randomly chosen 20% of the BHAs) and no intervention (1) generates comparatively high incidence (Fig. 4a, left, red) and autocorrelation levels (Fig. 4b, left, red). This is followed by a quick decay and a second, smaller peak, before the end of the outbreak. When a great number of starting locations exist (the seed is to infect 0.1% of the population in randomly chosen 60% of the BHAs) (2), incidence levels and autocorrelation patterns are similar, but autocorrelation levels are considerably lower (Fig. 4b, left, green).

We also observe that when the system is modified by external intervention, the shape of Moran’s I evolution changes dramatically with respect to the baseline level. Specifically, when mobility is 100 times lower (3) the rise of the incidence curve (Fig. 4a, right, orange) is not followed by Moran’s I, which in turn peaks when the incidence is already decaying (Fig. 4b, right, orange). Conversely, when the mobility is left unchanged but the transmissibility of the virus is reduced (4), Moran’s I evolution resembles the baseline one albeit with a lower maximum value and a slower pace (Fig. 4b, right, blue). Lastly, a very early total shutdown of mobility together with reduced transmissibility (5) can destroy the spatial autocorrelation (Fig. 4b, right, black).

## Discussion

In this research, the evolution of the COVID-19 pandemic in Catalonia has been first described in a wide period and at a small area level, paying particular attention to the comparison between the successive waves occurring within the study period. Few studies have considered such a wide period and therefore are capable of comparing these dynamics on multiple waves.

From the epidemic management point of view, the dissonance between wave declaration and the generalized increase of R(t) over the threshold value of 1 is a clear indicator of the reactive strategy followed by most European governments of “living with the virus”. Certainly, in contrast to some Asian countries and, particularly, China, who have followed a more proactive strategy of complete suppression of the virus, this strategy focused on preventing the collapse of the health system rather than curtailing the transmission. In fact, to reach the highest alert level in Spain it was necessary to have at least two indicators related to incidence at their highest risk and, at the same time, at least one related to hospitalization. This explains why waves and measures were not directly attached to epidemic growth -as measured by R(t)- but to already very high incidence levels (due to the lag between contagion and hospitalization).

The use of R(t) for real-time epidemic management is problematic because of data quality issues. In fact, R(t) was one of the indicators proposed by the Spanish authorities to measure the evolution of the epidemic, but it was considered an auxiliary indicator not directly related to the alert level[^11^]. Yet, it seems clear that, even if the surveillance system is not good enough to provide reliable estimates of R(t) in real-time, this measure should be used to properly define the beginning of a wave in retrospective analysis, even if incidence might grow very slowly when basal incident taxes are low.

Description shows the strong synchronic component of the outbreaks, probably explained by the explosive capacity of transmission of the virus in a comparatively small and highly integrated territory as Catalonia. As a consequence, it is not easy to discern spatial patterns and each wave probably has its specificities. The former is also likely to induce quick changes in spatial autocorrelation. While global spatial autocorrelation is almost always significant over the period, their values vary wildly within and between waves, and the time series pattern is roughly similar independently of the proximity criteria considered.

We propose plausible mechanisms for the qualitative form of autocorrelation evolution within each wave that are reinforced by the results of simulation. First, baseline evolution characterized by peaks of (comparatively) high autocorrelation in the first weeks of a wave is probably caused by local outbreaks that spread over geographically adjacent BHAs. As the outbreak reaches further areas and R(t) falls below 1 because of interventions, incident cases evolution becomes more independent of surrounding areas and, as a consequence, spatial autocorrelation quickly descends.

However, some waves differ from this baseline pattern, either because autocorrelation peaks are much lower (4th wave and 2nd phase of the 2nd wave) or differ more widely from it (3rd wave). Simulation results indicate that many independent focuses of the disease (as may happen when the risk of regrowth is high in sparse areas) reproduce the baseline pattern at lower autocorrelation levels, while interventions limiting human mobility and virus transmission can alter both autocorrelation levels and patterns. In fact, the strongest NPIs were ongoing during the third wave. Likely, high vaccination levels should also be counted among interventions “distorting” what we call the baseline pattern.

The results of this research have some limitations. We have privileged the analysis of successive waves in a single territory, while our conclusions would have benefited from a comparative perspective between territories. As it is well known, spatial analysis results are frequently dependent on the spatial units chosen. Although we have data for relatively small areas, we make assumptions of equal density inside these BHAs, which in some less populated areas of the region fail to correctly describe the heterogeneous distribution of population in space. Finally, the simulation models could have incorporated more realistic assumptions to bring their behavior closer to the observed case.

Notwithstanding the described obstacles, we have provided evidence that spatial autocorrelation values concerning COVID-19 incidence are inherently contingent on the outbreak phase, while they are also substantially affected by any external intervention affecting human behavior imposed on the system. These results should be taken into account by researchers looking for some spatial and spatio-temporal logic in COVID-19 (and more generally epidemic diseases) outbreaks.

## Supplementary Information

Supplementary Information.

## Data Availability

This research is based on open data. The raw data on COVID-19 incidence are available from ‘Record of COVID-19 tests performed in Catalonia. Segregation by gender and ABS’, https://analisi.transparenciacatalunya.cat/Salut/Registre-de-casos-de-COVID-19-a-Catalunya-per-rea-/xuwf-dxjd, last retrieved on 2022/03/17. Population with the right to receive health care from public financing in Catalonia by BHA can be obtained from this source: https://analisi.transparenciacatalunya.cat/Salut/Registre-central-de-poblaci-del-CatSalut/ftq4-h9vk/data.qety46t. BHA’s shapefiles are available at https://salutweb.gencat.cat/ca/el_departament/estadistiques_sanitaries/cartografia, April 2021 file.Raw mobility data were obtained from https://www.mitma.gob.es/ministerio/covid-19/evolucion-movilidad-big-data/opendata-movilidad, consulted 2022/03/28. Finally, vaccination levels evolution in Catalonia were be consulted here: https://www.idescat.cat/indicadors/?id=conj&n=14357, consulted 2023/01/13.
