# Supplementary material for: Key epidemiological indicators and spatial autocorrelation patterns across five waves of COVID-19 in Catalonia
Source: Sci Rep. 2023 Jun 15;13:9709. doi: 10.1038/s41598-023-36169-2 (PMC10272129; doi:10.1038/s41598-023-36169-2)
Supplement: Supplementary file 1 — Supplementary Information. [file 41598_2023_36169_MOESM1_ESM.docx]

## Supplementary information


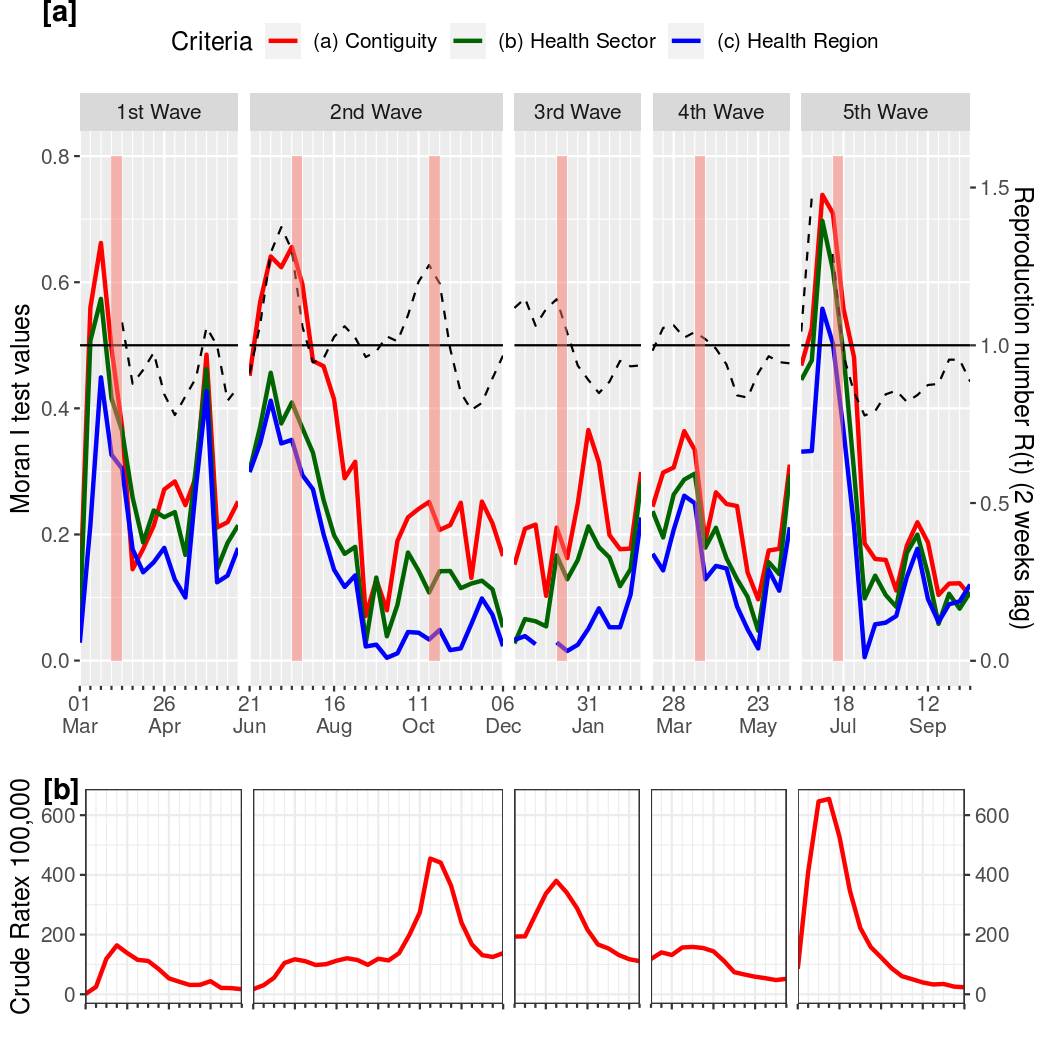


**Figure A1** - Catalonia global spatial autocorrelation values (Moran’s I) of weekly crude incidence rates according to Contiguity, Health Sector and Health Region membership (left axis), and effective reproduction number R(t) (right axis) [a], plus Catalonia crude incidence rate evolution [b]. Incidence peaks weeks are marked as reddish vertical lines in Figure A1[a]. Note: This Figure was created by the authors within the R environment using the ggplot2 package v. ‘3.4.0’, <https://ggplot2.tidyverse.org>.

*
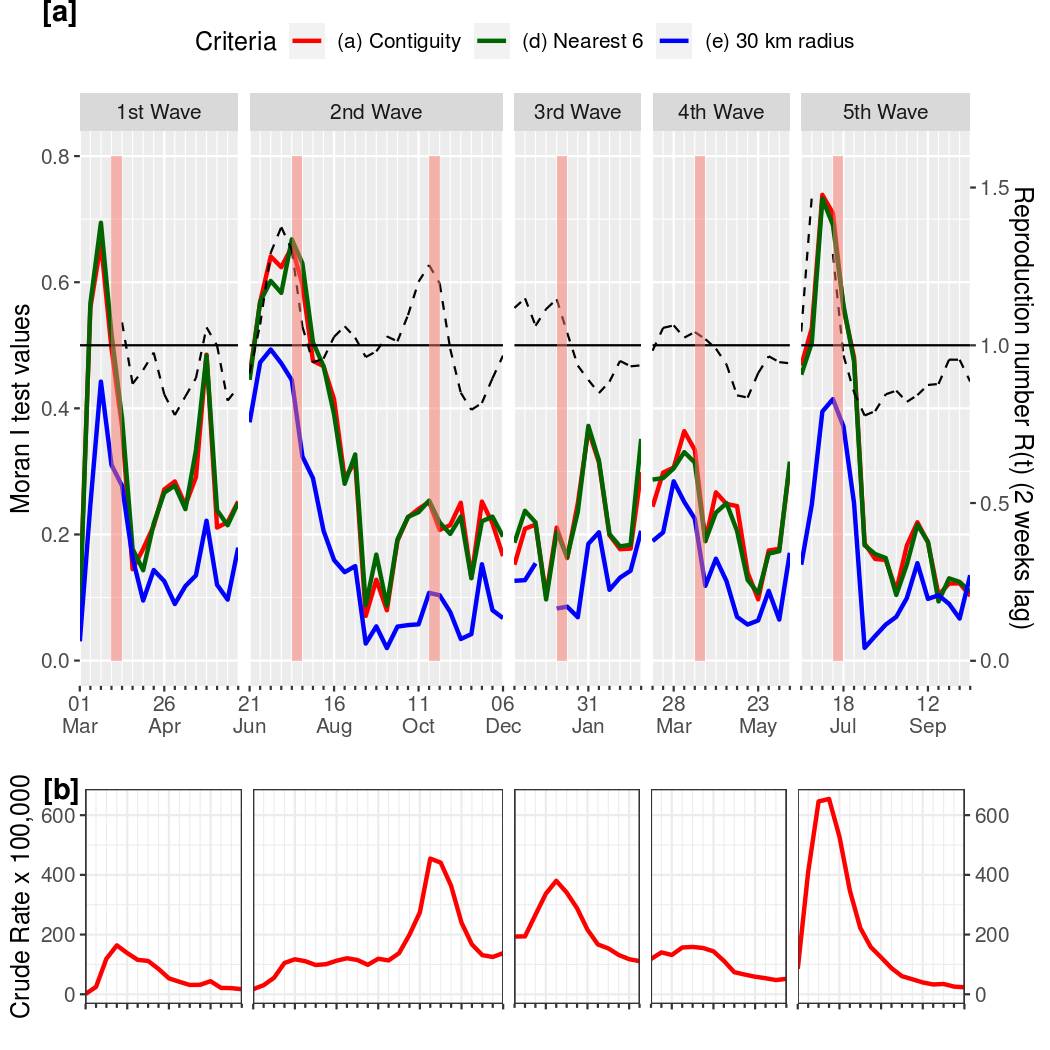
*

**Figure A2** - Catalonia global spatial autocorrelation values (Moran’s I) of weekly crude incidence rates according to Contiguity, 6-nearest neighbors and 30 km radius proximity criteria (left axis), and effective reproduction number R(t) (right axis) [a], plus Catalonia crude incidence rate evolution [b]. Incidence peaks weeks are marked as reddish vertical lines in Figure A2[a]. Note: This Figure was created by the authors within the R environment using the ggplot2 package v. ‘3.4.0’, <https://ggplot2.tidyverse.org>.

**Table A1. *Results of linear regression models predicting local Covid-19 crude incidence rates***

|  |  |  |  |  |  |  |  |  |  |
| --- | --- | --- | --- | --- | --- | --- | --- | --- | --- |
|  |  | Model | | | | | | | |
| Coef. |  | *[a] Empty Model* | *p* |  | *[b] Temp.Lagged incidence* | *p* |  | *[c] Spatiotemp. Lagged incidence* | *p* |
|  |  |  |  |  |  |  |  |  |  |
| Intercept |  | 150.734 | <0.001 |  | 34.614 | <0.001 |  | 23.707 | <0.001 |
| Lagged Incidence |  | --- | --- |  | 0.775 | <0.001 |  | --- | --- |
| Spatiotemp. Lag |  | --- | --- |  | --- | --- |  | 0.849 | <0.001 |
|  |  |  |  |  |  |  |  |  |  |
| R-squared |  | --- |  |  | 0.601 |  |  | 0.564 |  |
| d.f. |  | 31534 |  |  | 31162 |  |  | 31162 |  |
| Residual Std. Error |  | 165.4 |  |  | 104.5 |  |  | 109.3 |  |
|  |  |  |  |  |  |  |  |  |  |

**Table A2. *Results of linear regression models predicting local Reproduction number (Rt)***

|  |  |  |  |  |  |  |  |  |  |
| --- | --- | --- | --- | --- | --- | --- | --- | --- | --- |
|  |  | Model | | | | | | | |
| Coef. |  | *[a] Empty Model* | *p* |  | *[b]  Lagged Stringency* | *p* |  | *[c] [b]+Vaccinated Pop. %* | *p* |
|  |  |  |  |  |  |  |  |  |  |
| Intercept |  | 1.007 | <0.001 |  | 1.325 | <0.001 |  | 1.767 | <0.001 |
| Stringency Index^1^ |  | --- | --- |  | -0.005 | <0.001 |  | -0.011 | <0.001 |
| Vaccinated Pop. (%)^2^ | | --- | --- |  | --- | --- |  | -0.005 | <0.001 |
|  |  |  |  |  |  |  |  |  |  |
| R-squared |  | --- |  |  | 0.103 |  |  | 0.323 |  |
| d.f. |  | 31107 |  |  | 31106 |  |  | 31105 |  |
| Residual Std. Error |  | 0.2016 |  |  | 0.1909 |  |  | 0.1659 |  |
|  |  |  |  |  |  |  |  |  |  |
| ^1^ Lagged Stringency Index for Spain | | |  |  |  |  |  |  |  |
| ^2^ Percentage of vaccinated population (two doses) | | | | | |  |  |  |  |


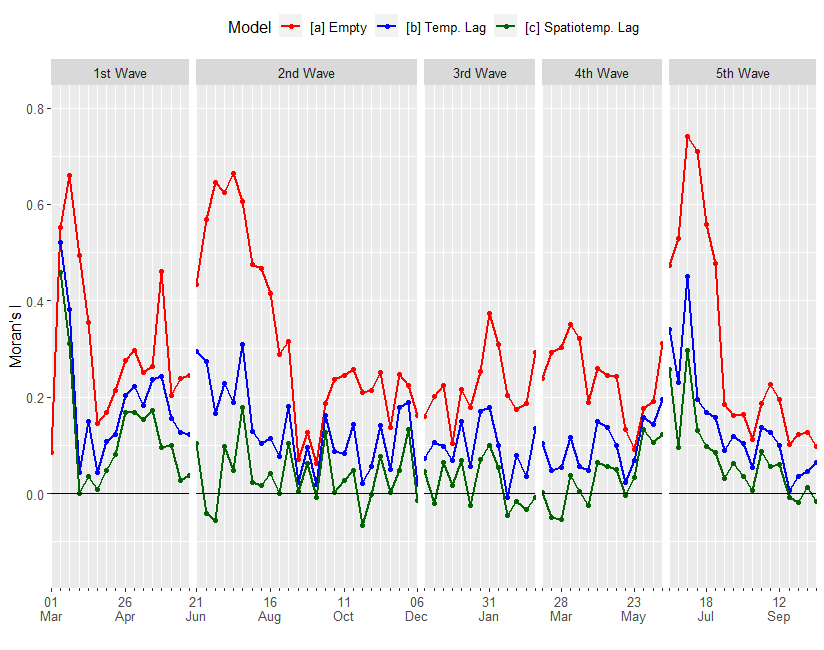


**Figure A3** - Catalonia global spatial autocorrelation values (Moran’s I) calculated from weekly residuals of linear regression models predicting crude incidence rate: [a] Empty model; [b] Temporal lag of the BHA used as a predictor and [c] Lagged mean crude incidence rate of the contiguous BHAs (spatiotemporal lag) used as a predictor). Note: This Figure was created by the authors within the R environment using the ggplot2 package v. ‘3.4.0’, <https://ggplot2.tidyverse.org>.
